# *Notes from the Field*: Fatalities Associated with Human Adenovirus Type 7 at a Substance Abuse Rehabilitation Facility — New Jersey, 2017

**DOI:** 10.15585/mmwr.mm6712a6

**Published:** 2018-03-30

**Authors:** Faye Rozwadowski, Mardea Caulcrick-Grimes, Lisa McHugh, AnnMarie Haldeman, Tara Fulton, Marie Killerby, Eileen Schneider, Xiaoyan Lu, Senthilkumar K. Sakthivel, Julu Bhatnagar, Demi B. Rabeneck, Sherif Zaki, John Watson

**Affiliations:** ^1^Epidemic Intelligence Service, CDC; ^2^New Jersey Department of Health; ^3^Respiratory Viruses Branch, Division of Viral Diseases, National Center for Immunization and Respiratory Diseases, CDC; ^4^Infectious Diseases Pathology Branch, Division of High Consequence Pathogens and Pathology, National Center for Emerging and Zoonotic Diseases, CDC.

On February 3, 2017, a local health department notified the New Jersey Department of Health (NJDOH) of a severe respiratory illness outbreak, including two hospitalizations and one death, at a substance abuse treatment facility. During December 2016–January 2017, NJDOH surveillance for noninfluenza respiratory viruses identified multiple human adenovirus (HAdV) cases in the surrounding community. HAdVs can cause severe respiratory illness, and outbreaks of HAdV type 4 (HAdV-4) and HAdV type 7 (HAdV-7) have been associated with communal living facilities, including military barracks (*1*). A combined HAdV-4 and HadV-7 live oral vaccine is available but is currently limited to military use (*2*). NJDOH and the local health department investigated the outbreak in consultation with CDC to describe outbreak scope and provide infection control recommendations in this communal facility.

The facility has an average outpatient census of 25 persons, an average daily census of 85 inpatients, and 91 staff members. Both staff members and patients congregate in multiple communal areas for group therapy sessions, recreational and social activities, smoking, and eating. In this outbreak, a probable case was defined as the occurrence of an acute respiratory illness (defined as any two of the following: fever ≥100°F [37.8°C], sore throat, cough, rhinorrhea, or nasal congestion) in a patient with an epidemiologic link to the treatment facility during January 1, 2017–March 31, 2017. Confirmed cases met the probable case definition and had a positive test result for HAdV using polymerase chain reaction (PCR) on a nasopharyngeal swab, oropharyngeal swab, or lung tissue specimen. Seventy-nine probable cases including 59 inpatients and 20 staff members were identified. Among these 79 patients, four (5%) were hospitalized, and three died (case fatality rate 4%). Specimens were available from 25 probable cases; four of these, all in hospitalized patients, were confirmed as HAdV by PCR. The three fatal cases included two patients with HAdV-7 identified from nasopharyngeal specimens and one with HAdV-7 identified from a lung tissue specimen at autopsy. HAdV detected from the fourth patient was not typed.

The three persons who died initially developed fever and cough, which rapidly progressed to multifocal pneumonia and acute respiratory distress syndrome that required intubation and mechanical ventilation. Respiratory failure progressed and required extracorporeal membrane oxygenation; respiratory failure was followed by acute renal failure and death. Among the three fatal cases, time from symptom onset to death ranged from 4 to 37 days; patients ranged in age from 54 to 64 years, and two were men. According to the patients’ medical histories, one had cirrhosis, one had diabetes mellitus type 2, and one had both cirrhosis and diabetes mellitus type 2. All three deaths occurred in persons who reported a history of alcoholism. Alcohol abuse independently increases the risk for acute respiratory distress syndrome approximately threefold to fourfold (*3*).

The outbreak setting presented challenges in management and control of HAdV transmission because of the communal living and group-therapy environment. Local, state, and federal officials recommended 1) use of U.S. Environmental Protection Agency-approved viricide cleaners on common touch areas in communal gathering places, 2) frequent patient and staff member handwashing, 3) isolation of patients with fever ≥100°F (37.8°C) lasting ≥24 hours, and 4) a 72-hour deferral for new admissions during implementation of recommended infection control measures. No new cases were reported after March 24, 2017.

HAdV-7 is known to cause morbidity and mortality, particularly in military training facilities (*4*). Adenovirus morbidity and mortality associated with nonmilitary congregate settings are less well described, although severe morbidity and mortality have been documented among immunocompromised patients (*5*). This outbreak investigation documents severe morbidity and mortality associated with HAdV-7 among persons in a substance abuse treatment facility with specific comorbidities including diabetes mellitus type 2, alcoholism, and cirrhosis and highlights the challenges of illness containment in a communal environment. Clinicians and public health practitioners should be aware of HAdV-7 as a potential cause of severe respiratory illness in these settings.
